# Supplementary material for: Using old antibiotics to treat ancient bacterium—β-lactams for Bacillus anthracis meningitis
Source: PLoS One. 2020 Feb 13;15(2):e0228917. doi: 10.1371/journal.pone.0228917 (PMC7018077; doi:10.1371/journal.pone.0228917)
Supplement: S1 Appendix — (DOCX) [file pone.0228917.s001.docx]

Supplement 1. Pharmacokinetics of Meropenem following IV injection of 40 mg/kg or 150 mg/kg to rabbits.

Rabbits were injected IV with 40 or 150 mg/kg of Meropenem (two rabbits per dose). Inhibitory concentration of the antibiotic in the serum was tested at pre-injection and 0.5, 1, 2, 4, and 6 h post antibiotics injection. The minimal serum inhibitory concentration was determined as the highest dilution that inhibited *Bacillus anthracis* Vollum strain growth. The Serum Inhibitory Concentration is presented as 1/X of the dilution.
