# Supplementary material for: Using old antibiotics to treat ancient bacterium—β-lactams for Bacillus anthracis meningitis
Source: PLoS One. 2020 Feb 13;15(2):e0228917. doi: 10.1371/journal.pone.0228917 (PMC7018077; doi:10.1371/journal.pone.0228917)
Supplement: Attachment — Submitted filename: Response to Reviewers final.docx [file pone.0228917.s002.docx]

PONE-D-19-30028
Using old antibiotics to treat ancient bacterium - β-lactams for Bacillus anthracis meningitis
PLOS ONE

Dear Dr. Haroon Mohammad,

Enclosed pleas find revised version of all the figures, according to the reviewers request. We would like to thank the reviewers for careful reading of the manuscript and making helpful remarks.

Hope you will find the revised version of this manuscript suitable for publication in PLOS one.

Yours

Haim Levy
